# Pituitary Adenylate Cyclase Activating Peptide Deficient Mice Exhibit Impaired Thymic and Extrathymic Regulatory T Cell Proliferation during EAE

**DOI:** 10.1371/journal.pone.0061200

**Published:** 2013-04-16

**Authors:** Yossan-Var Tan, Catalina Abad, Yuqi Wang, Robert Lopez, James A. Waschek

**Affiliations:** Semel Institute/Department of Psychiatry, David Geffen School of Medicine, University of California Los Angeles, Los Angeles, California, United States of America; Escola Paulista de Medicina - UNIFESP, Brazil

## Abstract

We have shown that mice deficient in pituitary adenylate cyclase-activating polypeptide (PACAP, gene name ADCYAP1) manifest enhanced sensitivity to experimental autoimmune encephalomyelitis (EAE), supporting the anti-inflammatory actions described for this neuropeptide. In addition to an increased proinflammatory cytokine response in these mice, a reduction in regulatory T cell (Treg) abundance in the lymph nodes (LN) was observed, suggesting altered Treg kinetics. In the present study, we compared in PACAP deficient (KO) vs. wild type mice the abundances and rates of proliferation FoxP3^+^ Tregs in three sites, the LN, central nervous system (CNS) and thymus and the relative proportions of Th1, Th2, and Th17 effector subsets in the LN and CNS. Flow cytometry analyses revealed a decrease in Treg proliferation and an increased T effector/Tregs ratio in the LN and CNS of PACAP KO mice. In the thymus, the primary site of *do novo* natural Treg production, the total numbers and proliferative rates of FoxP3^+^ Tregs were significantly reduced. Moreover, the expression of IL-7, a cytokine implicated in thymic Treg expansion during EAE, failed to increase at the peak of the disease in the thymus and LN of PACAP KO mice. In addition to these Treg alterations, a specific reduction of Th2 cells (about 4-fold) was observed in the lymph nodes in PACAP KO mice, with no effects on Th1 and Th17 subsets, whereas in the CNS, Th1 and Th17 cells were increased and Th2 decreased. Our results suggest that endogenous production of the neuropeptide PACAP protects against EAE by modulating Treg expansion and Th subsets at multiple sites.

## Introduction

It has been shown that the nervous system and the immune system interact with each other during health and disease. In this regard, there is robust evidence that neurological mediators, including multiple neurotransmitters and neuropeptides, exert modulatory effects on immune cells such as myeloid cells or lymphocytes, which are key players of innate and adaptive immunity. Understanding how the nervous system regulates the immune system is crucial to comprehend the complex pathogenesis of autoimmune diseases, and to develop new therapeutic tools. A neuropeptide with well-described modulatory actions in the nervous, endocrine and immune systems is pituitary adenylate cyclase-activating polypeptide (PACAP, gene name ADCYAP1) [1]. It binds to three receptors of the G-protein coupled receptor (GPCR) family, VPAC1, VPAC2 and PAC1, which are present in the surface of various cell types including many immune cell types [2], [3]. The names of these receptors refer to their ligand affinities: whereas the VPAC receptors bind both PACAP and the highly-homologous polypeptide vasoactive intestinal peptide (VIP) with similar affinity, PAC1 is a PACAP-preferring receptor. These receptors stimulate a canonical adenylate cyclase (AC)/cyclic AMP (cAMP)/protein kinase A (PKA) signalling pathway, but can in a variety of contexts activate inositol triphosphate/PLC/PKC and other signalling pathways [4], [5]. Although PACAP exerts diverse actions in the immune system, it is primarily recognized as an anti-inflammatory peptide. In this respect, PACAP strongly inhibits the release of proinflammatory cytokines such as TNFα, IL-6 and IL-12 and chemokines such as RANTES, KC, MIP-1α, MIP-1β and MCP-1 from macrophages and primary microglia stimulated *in vitro* with lipopolysaccharide (LPS) [2], [3]. Evidence suggest that these effects are mediated at least in part by activation of PKA, but also by inhibiton of NF-kB and/or MEKK1/MEK4/JNK pathways, and by induction of CREB phosphorylation [5]. In addition, it has been shown that PACAP modulates T cell function, promoting Th2 over Th1/Th17 cytokine profiles [2], [3]. In this respect, PACAP acts on antigen presenting dendritic cells and macrophages by promoting the production of purported Th2-recruiting chemokines (CCL11 and CCL22), modifying the expression of co-stimulatory molecules (B7.1 and B7.2), and promoting the generation of Th2 vs Th1 memory cells [6]–[9]. The anti-inflammatory and Th2-promoting actions of PACAP have been corroborated by studies using *in vivo* models of acute and chronic inflammation, including experimental autoimmune encephalomyelitis (EAE), which exhibits many of the clinical and molecular features of multiple sclerosis (MS) [10]. Moreover, PACAP and/or PACAP mRNA have been shown to be strongly induced in neurons in several models of inflammation [11]–[14], and have been found to be also expressed by lymphocytes in naïve animals [15], [16].

In chronic inflammatory diseases such as MS, a complex interplay between Th1, Th17, Th2 and regulatory T cells (Tregs) is believed to determine the evolution and outcome of the disease [17]. In the EAE model induced with MOG (myelin oligodendrocyte glycoprotein), MOG-responsive Th cells are generated in the draining lymph nodes located close to the immunization site. Then, T cells migrate to the CNS, where they exert their pro or antiinflammatory actions. For example, Th1 and Th17 cells produce cytokines that primarily sustain the proinflammatory activities of macrophages, whereas Th2 cells and Tregs generally limit inflammation. With respect to the latter, Tregs have been classified as natural (nTregs) and inducible or adaptive (iTregs), and are characterized by the expression of CD25 and the transcription factor forkhead box P3 (Foxp3) [18]. Whereas nTregs become fully differentiated in the thymus, iTregs can be generated in the periphery from naïve T cells under chronic inflammatory conditions [19], and can be generated *in vitro* from uncommitted naïve T cells stimulated by antigen under the influence of immunosuppressive cytokines (IL-10 and TGF-β). It has been shown that Tregs accumulate in the CNS as EAE progresses and may promote recovery from the disease by inhibiting the activity of autoreactive T cells and secreting IL-10 and TGF-β [20]. Korn *et al* [21] demonstrated that in the model of EAE in C57BL/6 mice induced with MOG_35–55_, Tregs are generated *via* expansion of the pre-existing FoxP3^+^ nTreg population, rather than *de novo* from non-Tregs (FoxP3-negative). Another study demonstrated that nTreg proliferation increased dramatically in the thymus at the peak of EAE and was maintained throughout the recovery phase of the disease [22]. In parallel, an induction in IL-7 expression was found in the thymus, and mice treated with an IL-7 blocking antibody displayed a significant decrease in thymic Treg proliferation, suggesting a critical regulatory role for this cytokine for Treg expansion during EAE [22]. On the other hand, an increase in Treg numbers in the CNS during the recovery phase of the disease has been ascribed to local proliferation of these cells driven by inflammation [23]. Because of the putative beneficial effects of Tregs, understanding the dynamics of this cell population and the discovery of agents that promote their expansion may be critical to develop new therapeutic strategies against inflammatory autoimmune diseases such as MS.

We have previously reported that PACAP KO mice exhibit exacerbated EAE, which occurred in association with increased proinflammatory cytokine gene expression in the inflamed CNS [24]. In addition, we found that the abundance of Tregs was diminished in the lymph nodes (LN) of PACAP deficient mice on day 20 post-EAE induction, suggesting an alteration in Treg homing, proliferation, or survival. In the present work, we measured the abundance of Tregs at multiple sites, and studied their extent of proliferation by flow cytometry. We observed reduced rates of FoxP3^+^ Treg proliferation and higher Teff/Treg ratios in the lymph nodes and CNS of PACAP KO mice. In addition, the total numbers and proliferative rates of Tregs were significantly reduced in the thymi of PACAP KO mice. Flow cytometry analysis of Th profiles revealed a reduction in the Th2 cell subset in the lymph nodes and CNS of PACAP KO mice. Thus, our results suggest that endogenous PACAP protects against EAE by supporting nTreg proliferation and Th2 subsets.

## Materials and Methods

### Ethics Statement

Animal studies were approved by the institutional animal research committee of the University of California at Los Angeles (UCLA).

### EAE Induction and Scoring System

Eight to 12 week old female WT or PACAP KO mice on a C57BL/6 background (backcrossed for at least 12 generations) were used in all experiments. For EAE induction, an emulsion consisting on 50 µl of PBS containing 100 µg of MOG_35–55_ (GLBiochem, Shanghai, China) and 50 µl of Complete Freund’s Adjuvant (Difco, Detroit, MI) supplemented with 5 mg/ml *Mycobacterium tuberculosis* (H37Ra, Difco), were injected subcutaneously in the two flanks of mice under light anesthesia. Mice received an additional intraperitoneal injection of 200 ng of pertussis toxin (List Biological Laboratories, Campbell, CA) in PBS on days 0 and 2 post-immunization. Mice were monitored daily for symptoms of EAE as described [24] and all efforts were made to minimize suffering. Briefly, a score of 0 corresponds to asymptomatic mice, 1, to mice with no tail tone, 2, to mice with wobbling gait, 3, to mice with hind limb paralysis and 4, to moribund or dead mice.

### Cell Suspension Preparation

Thymi, axillary lymph nodes and brains and spinal cords were dissected from mice previously perfused with PBS. For preparation of thymus and lymph node single cell suspensions, organs were tapped through a 40 µm nylon cell strainer. For CNS mononuclear cell suspension preparation, brain and spinal cords were excised in small fragments, and incubated for 45 min at 37°C in HBSS containing 0.1 mg/ml of DNAse I (Worthington Biochemical Corporation, Lakewood, NJ) and 0.1 mg/ml of collagenase IV (Roche, Indianapolis, IN). For further dissociation of the tissues, solutions were passed several times through a 18 gauge needle. The CNS homogenate was filtered through a 40 µm mesh, and cells were pelleted by centrifugation. Pellets were resuspended in 5 ml of 40% Percoll (GE Healthcare, Piscataway, NJ), underlayered with 5 ml of 80% Percoll and centrifuged for 30 min at 500 g with no brake. CNS mononuclear cells were obtained from the interface of the 40/80% Percoll gradient.

### Flow Cytometry

To study Treg proliferation, cells were stained for 15 min at 4°C with PerCP-Cy5.5-anti-CD4 and PE-anti-CD25 antibodies (eBioscience) for lymph nodes and CNS, and PerCP-Cy5.5-anti-CD4 and PE-anti-CD8 antibodies (eBioscience) for thymus, and then fixed and permeabilized at 4°C O/N with fixation/permeabilization buffer (eBioscience). After two washes with PBS, cells were incubated with IgG blocking solution (eBioscience) for 15 min at 4°C and then with APC-anti-Foxp3 (eBioscience) and FITC-anti-Ki67 (BD Biosciences) antibodies for 2 hours at 4°C in permeabilization buffer (eBioscience). Fluorescence minus one (FMO) controls were used for gating purposes (Fig. S1).

For cytokine intracellular staining, cells were incubated in RPMI with 2% fetal bovine serum (FBS) and 1% penicillin/streptomycin and with 50 ng/ml PMA (Sigma-Aldrich, St Louis, MO), 1 mg/ml of ionomycin (Sigma-Aldrich) and 1X brefeldin and 1X monensin (eBioscience, San Diego, CA) for 4 hours at 37°C. After surface staining with FITC-anti-CD4 antibody (eBioscience), cells were fixed for 15 min with 2% PFA and then permeabilized with PBS/0.2% Tween 20. Then, cells were incubated with PE-anti-IFNγ, APC-anti-IL-4 and PerCPCy5.5-anti-IL-17 antibodies in PBS/0.2% Tween 20/5% FBS/2% BSA for 1 h at 4°C at the dilutions recommended by the manufacturer (eBioscience). Unstimulated samples were used as controls for gating purposes (Fig. S2).

For all experiments, samples were acquired with a FACScalibur instrument (BD Biosciences), and analyzed using the Weasel software (Walter and Eliza Hall Institute for Medical Research, Melbourne, Australia).

### Real Time RT-PCR

RNA was extracted using the Trizol reagent (Sigma) according to the manufacturer’s protocol. RNA samples were treated with DNase I (DNA-free™ kit, Ambion, Austin, TX) according to the manufacturer’s instructions to remove the genomic DNA. RNA was quantified by spectrophotometry (A_260/280 nm_), and 1 µg of total RNA was retrotranscribed with the Iscript kit from Bio-Rad (Hercules, CA). Real-time quantitative PCR assays were performed on an iCycler thermal cycler (Bio-Rad Laboratories, Hercules, CA) using iQ SYBR Green Supermix (Bio-Rad). Primers used were: for PACAP (GenBank accession number NM_009625), forward 5′-CCCCTTATTATTAGACTCTTACGGTG and reverse 5′-AGACAGTGACTGATGCTTCTCG-3′, for IL-7 (GenBank accession number: NM_008371), forward 5′-GCCTGTCACATCATCTGAGTGC-3′ and reverse 5′-TTCCTGTCATTTTGTCCAATTCA-3′ and for HPRT (GenBank accession number: NM_013556), forward 5′-TGGTGAAAAGGACCTCTCGAA-3′ and reverse 5′-TCAAGGGCATATCCAACAACA-3′. Amplification was performed as follows: for PACAP, initial denaturation at 95°C for 5 min, 40 cycles of denaturation at 95°C for 20 sec, annealing at 62°C for 30 sec and extension at 72°C for 20 sec, and final elongation at 72°C for 10 min; for IL-7, initial denaturation at 95°C for 5 min, 40 cycles of denaturation at 95°C for 1 min, annealing at 60°C for 1 min and extension at 72°C for 1 min, and final elongation at 72°C for 10 min. At the end of the PCR reaction, melting curve analysis was used to ensure the amplification of a single PCR product. The fold increase between sample A and sample B (control) in the cDNA (target gene) relative to the corresponding housekeeping gene (HPRT) was determined as follows: Fold increase  = 2^–ΔΔCt^, where ΔΔCt = (Ct_target_ – Ct_HPRT_)_sample A_ – (Ct_target_ – Ct_HPRT_)_sample B,_ where Ct denotes the threshold cycle of PCR amplification at which product is first detected by fluorescence.

### Statistical Analysis

The GraphPad Prism 4 program was used for statistical analysis and for generating graphs. ANOVA and Student’s *t*-est were used to assess significance.

## Results

### PACAP Gene Expression is Upregulated in the Spinal Cord and Lymph Nodes of WT Mice after Induction of EAE

**Figure 1:**
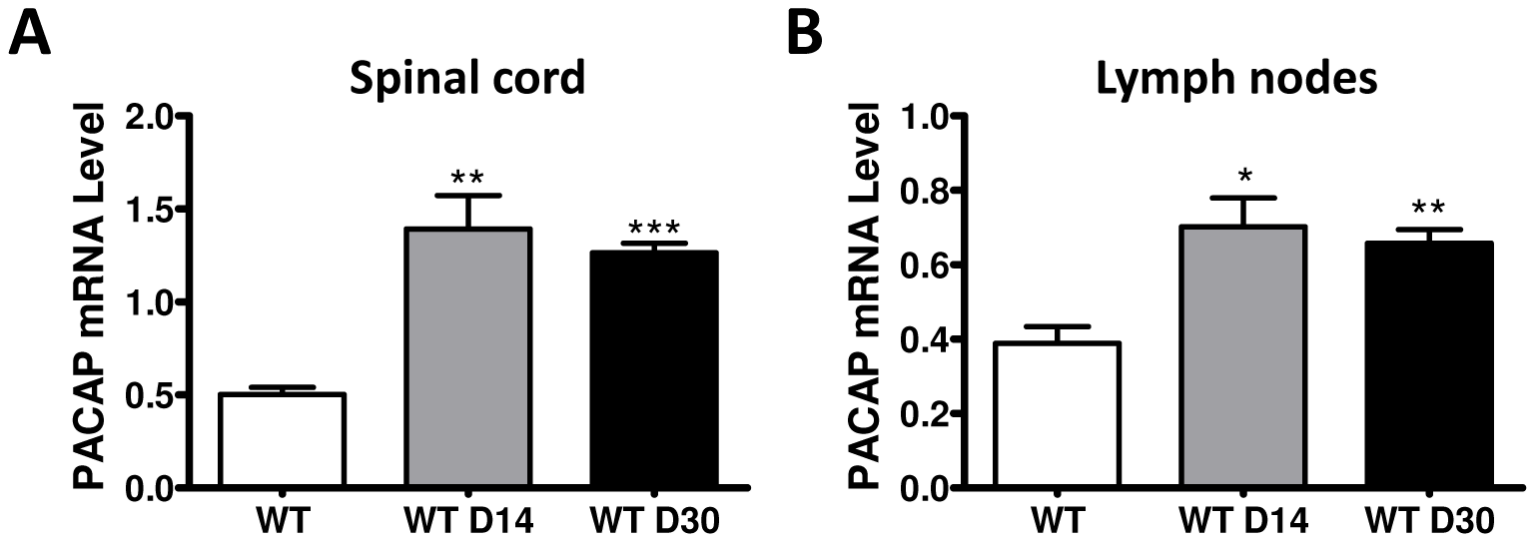
PACAP gene expression is induced in the spinal cord and the lymph nodes of WT mice during EAE. PACAP mRNA levels were determined by real time RT-PCR in the spinal cord (A) and the lymph nodes (B) of C57BL/6 mice on days 0, 14 and 30 post-disease induction. The expression of PACAP was elevated in both tissues on day 14, and maintained on day 30. Bar charts, mean ± SEM of six individual mice. Student’s *t*-test *P<0.05; **P<0.01; ***P<0.001.

PACAP is widely distributed in neurons of the CNS and periphery, and is expressed in lymphocytes and some endocrine cells [1]. Previous studies have reported a marked upregulation of PACAP mRNA and/or immunoreactivity in neurons and T cells in certain models of injury and/or inflammation, suggesting enhanced PACAP signalling and functional activity under these conditions [25], [26]. In order to determine if PACAP might be regulated in the CNS and immune cells in the EAE model, we examined PACAP gene expression at the peak of clinical disease and during recovery in the spinal cord, the main target tissue affected in this model of EAE, as well as in the draining lymph nodes and thymus. WT mice immunized with MOG exhibited a peak of EAE clinical symptoms approximately on day 14 followed by a spontaneous recovery [24], and as described before, PACAP KO mice exhibited exacerbated EAE (Fig. S3). We found that on day 14, the mRNA levels of PACAP in the spinal cord of WT mice were approximately 3-fold higher than those in uninduced mice (**P<0.01) (Fig. 1A). On day 30 post-EAE induction, PACAP mRNA expression remained upregulated, with only a minor reduction compared to day 14 (***P<0.001). Likewise, PACAP gene expression was found to be increased during EAE in the lymph nodes, although to a somewhat less degree that in the cord: it increased by almost 2-fold on day 14 (*P<0.05), and the levels decreased again slightly on day 30 (**P<0.01) (Fig. 1B). On the other hand, we found non-detectable levels of PACAP mRNA both before and after EAE in the thymus (data not shown).

### Treg Proliferation was Impaired in the Draining Lymph Nodes and CNS of PACAP KO Mice while T Effector/Treg ratios were Increased

**Figure 2:**
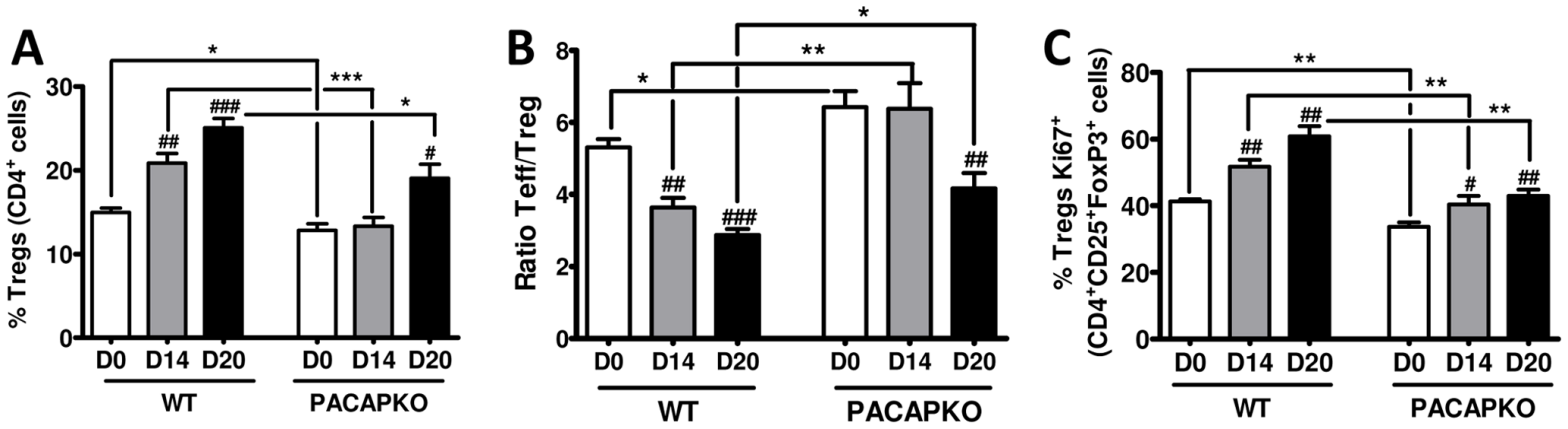
The relative abundance and proliferation of Tregs is diminished in the lymph nodes of PACAP KO and the ratio of Teff to Tregs is enhanced. Panel **A** indicates the percentages of CD4^+^ cells in the lymph nodes (LN) that were Tregs (CD4^+^CD25^+^Foxp3^+^) in WT and PACAP KO mice on days 0, 14 and 20 days after EAE induction. Panel **B** reports the ratio Teff/Treg (calculated as the % of CD4^+^CD25^+^ cells that are Fox3^−/^% of CD4^+^CD25^+^ cells that are Foxp3^+^). Panel **C** indicates, the percentage of CD4^+^CD25^+^Foxp3^+^ Tregs that are proliferating as determined by co-expression with the Ki67 antibody (C). Values represented are mean ± SEM. One experiment representative of three (n = 6 each) is shown. Student’s *t*-test *P<0.05; **P<0.01; ***P<0.001 (*for comparison between values of WT and PACAP KO) and ^#^ *P*<0.05; ^##^P<0.01; ^###^P<0.001 (^#^for comparison between values on 0, 14 or 20 days within WT or PACAP KO mice strains). See Fig. S4 for representative FACS plots and Fig. S5 for determinations of total number of cells, % of total cells that are Tregs, and total numbers of Tregs during the course of EAE in the lymph nodes of PACAP KO vs. WT mice.

**Figure 3:**
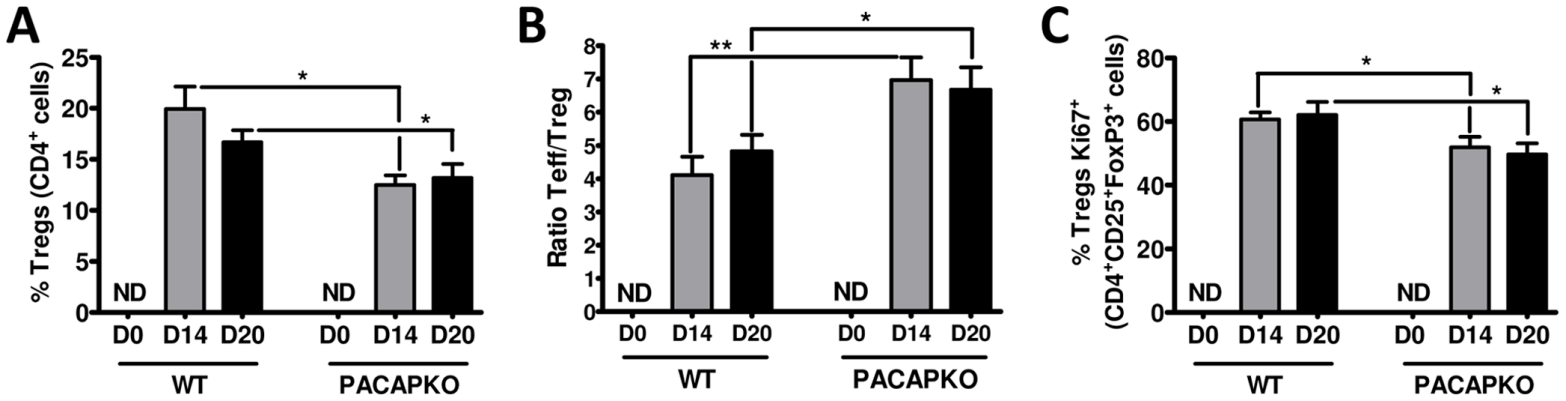
The relative abundance and proliferation of Tregs is diminished in the CNS of PACAP KO and the ratio of Teff to Tregs is enhanced. Panel **A** indicates the percentages of CD4^+^ cells in the CNS that were Tregs (CD4^+^CD25^+^Foxp3^+^) in WT and PACAP KO mice on days 0 (ND = non detectable), 14 and 20 days after EAE induction. Panel **B** reports the ratio Teff/Treg (calculated as the % of CD4^+^CD25^+^ cells that are Fox3^−/^% of CD4^+^CD25^+^ cells that are Foxp3^+^). Panel **C** indicates, the percentage of CD4^+^CD25^+^Foxp3^+^ Tregs that are proliferating as determined by co-expression with the Ki67 antibody (C). Values represented are mean ± SEM. One experiment representative of three (n = 6 each) is shown. Student’s *t*-test *P<0.05; **P<0.01. See Fig. S6 for representative FACS plots and Fig. S7 for determinations of total number of cells, % of total cells that are Tregs, and total numbers of Tregs during the course of EAE in the CNS of PACAP KO vs. WT mice.

Regulatory T cells are known to play crucial roles in limiting the development of autoimmunity, and promoting recovery from clinical disease. The primary locations where Tregs act to inhibit inflammation in EAE are still uncertain. However, it has been suggested that these cells restrict the priming of naïve T cells in the draining lymph nodes, but also suppress ongoing inflammatory responses in target tissues. We previously examined the presence of Tregs in the LN of WT and PACAP KO mice during the recovery period of EAE, and found that their relative abundance among CD4^+^ cells was reduced in the latter mice [24]. To gain a better understanding of this apparent Treg deficit, we compared in these mice the relative abundances, ratios of Teff/Tregs, and proliferative rates of Tregs at both the peak of disease and in the recovery period in the LN and also in the CNS. Proliferation of CD4^+^CD25^+^Foxp3^+^ Tregs was determined using the Ki67 marker, and expressed as percentage of CD4^+^CD25^+^Foxp3^+^ cells that were Ki67^+^. These studies revealed that the proportion of Tregs in the LN gated in the CD4^+^ T cell population was strongly reduced at both the peak (day 14) and recovery phase (day 20) of the disease (Fig. 2A and Fig. S4A and S5). Moreover, enhanced ratios of Teff/Tregs, which have been shown to correlate well with the severity of EAE [21], were observed in the LN of PACAP KO mice compared to WT mice at these time points (Fig. 2B). These changes were associated with a reduction in the percentage of CD4^+^CD25^+^Foxp3^+^ Tregs that were positive for Ki67 (Fig. 2C, and Fig. S4B), indicating a reduced level of Treg proliferation in the LN of PACAP-deficient mice. A qualitatively identical pattern of Treg parameters was observed in the CNS (Fig. 3A–C and Fig. S6). These results demonstrate that in the absence of endogenous PACAP, Treg proliferation during EAE was impaired and ratios of Teff/Tregs increased in both lymph nodes and CNS.

### Thymic Treg Proliferation and IL-7 mRNA Gene Expression is Impaired during EAE in PACAP KO Mice

**Figure 4:**
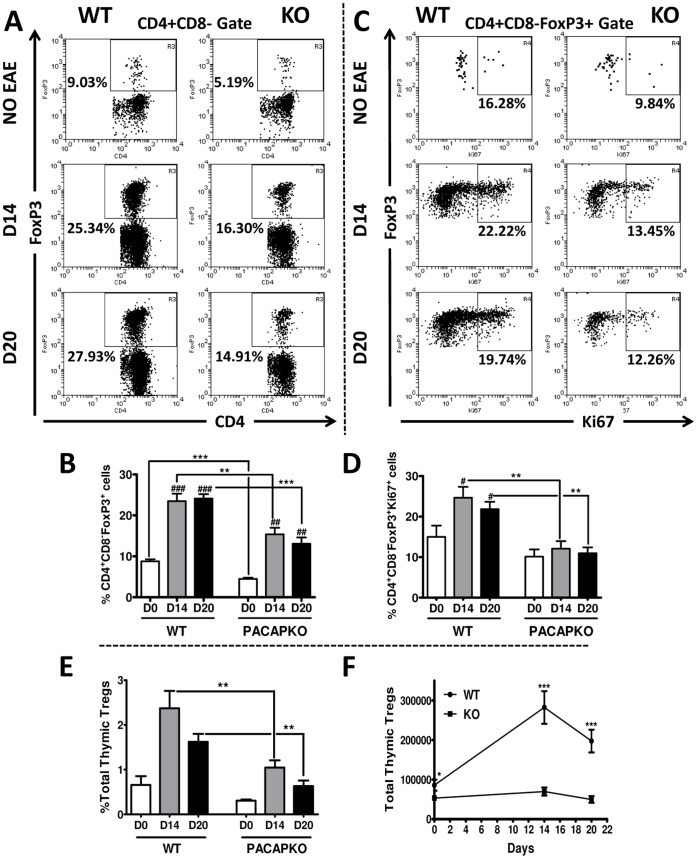
The expansion of thymic Tregs is impaired in PACAP KO mice. We determined the proportions (A, B, E) and total numbers (F) of Tregs in the thymus in mice with no EAE or mice with EAE on day 14 and 20 post-immunization by using CD4, CD8 and Foxp3 antibodies and proliferation (C, D) by using an anti-Ki67 antibody. **A** and **C** are representative FACS plots for each group, and **B** and **D** are the mean values ± SEM with Student’s *t*-test *P<0.05; **P<0.01; ***P<0.001 (*for comparison between values of WT and PACAP KO) and ^##^ *P*<0.01; ^###^ *P*<0.001 (^#^for comparison between values on 0, 14 or 20 days within WT or PACAP KO mice strains). Data shown are representative of three experiments. The Y axis in panel **B** represents the percentage of CD4^+^CD8^−^ cells that are Foxp3^+^. Panel **D**, the Y axis indicates the percentage of CD4^+^CD8^−^Foxp3^+^ cells that are Ki67^+^. See Fig. S8 for determinations of total number of cells during the course of EAE in the thymus of PACAP KO vs. WT mice.

It has been shown that the pool of Tregs expands during EAE and it has been suggested that nTregs, which develop in the thymus, should account for much of the enlarged population of Tregs in the periphery. Therefore we compared by flow cytometry the relative abundance, total numbers and proliferation rates of Foxp3^+^ Tregs in the thymus of PACAP KO vs WT mice on days 0, 14 and 20 after EAE induction (Fig. 4 and Fig. S8). We found that in uninduced animals, the proportion of thymic Tregs (defined as the proportion of CD4^+^CD8^−^ cells that were Foxp3^+^) was significantly lower in PACAP KO mice than in WT mice (***P<0.001) (Fig. 4A, B). Moreover, whereas an increase in Treg presence was found in the thymus of WT mice at the peak of the disease (14 days after MOG-immunization), the induction was significantly less pronounced in PACAP-deficient mice (Fig. 4A, B) (**P<0.01). A similar result was found at the recovery phase of the disease (20 days after EAE induction) with 24.1% of CD4^+^CD8^−^Foxp3^+^ Tregs in WT thymus vs 13.1% in PACAP KO thymus. In addition, the percentages of thymic cells that were Tregs (Fig. 4E) and the total numbers of thymic Tregs (Fig. 4F) were dramatically reduced in PACAP KO vs. WT mice at both the peak and recovery phase of disease. Interestingly, the total number of cells in the thymus was lower in PACAP KO mice than in WT mice on days 14 and 20 post-EAE induction (Fig. S8), perhaps due to the enhanced disease severity in these mice. The reduction in thymic Treg production could contribute to the decreased numbers of Tregs in PACAP KO lymph nodes and CNS. To examine this possibility, we measured by flow cytometry the proliferative index of Tregs in the thymus using the marker Ki67 as above. The proportion of proliferating Tregs (CD4^+^CD8^−^Foxp3^+^ cells that were Ki67^+^) in WT mice rose by 2-fold 14 days after EAE immunization, and slightly decreased on day 20, although it was still above basal levels (Fig. 4C and D). However, in the PACAP KO thymus, the Treg proliferative rate was lower than in the WT tissue in uninduced animals and proliferation did not increase with EAE. Therefore, our data suggests that impairment in Treg expansion in the PACAP KO thymus may also play a role in the reduction of Treg abundance and exacerbated EAE response of PACAP KO mice.

**Figure 5:**
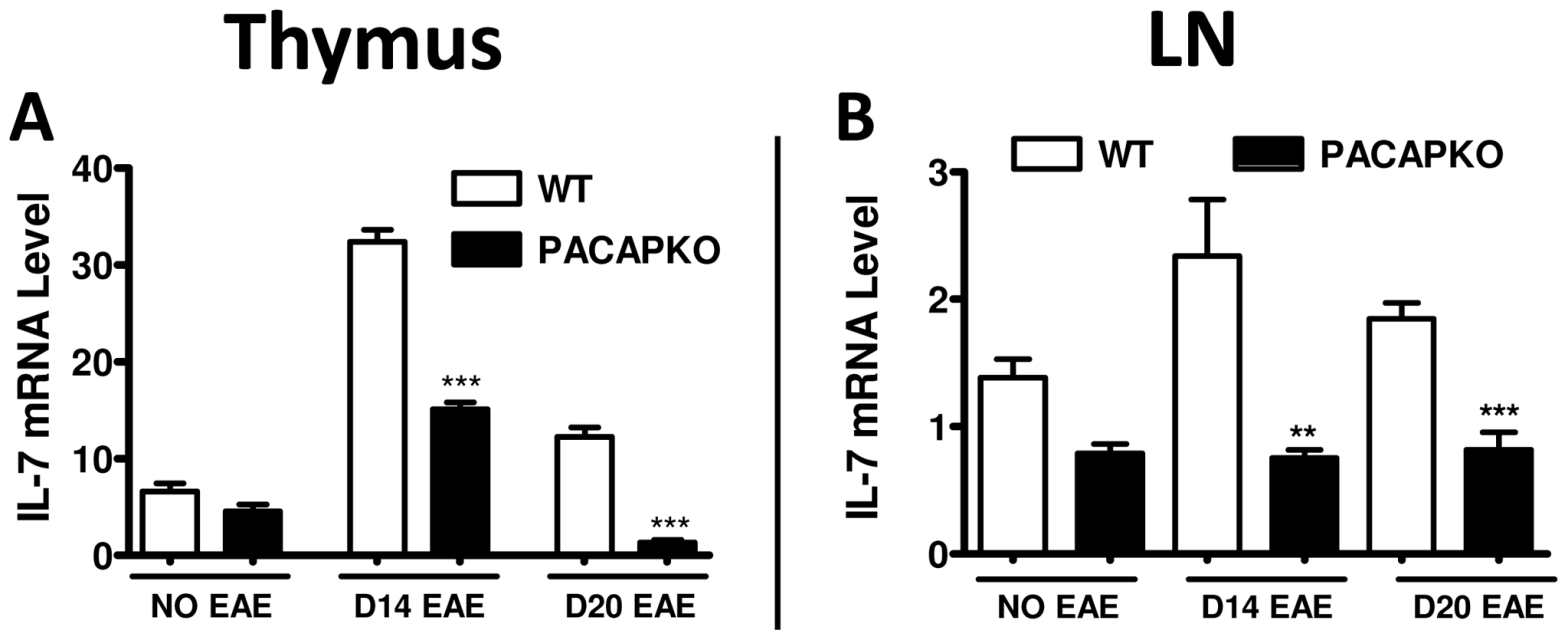
IL-7 gene expression in the thymus and lymph nodes of naive and MOG-injected WT and PACAP KO mice. The expressions of IL-7 mRNA in the thymus (A) and lymph nodes (LN) (B) of WT and PACAP KO mice were determined by real time RT-PCR on days 0, 14 and 20 after EAE immunization (n = 6 for each group). Arbitrary units were calculated using the 2^−ΔΔCt^ formula as described in Material and Methods and the means ± SEM are shown. All WT inductions were significant compared to basal levels. Significance of comparison between WT and PACAP KO at each time point analyzed is shown in the Fig., with **P<0.01; ***P<0.001 by Student’s *t-test.*

The mechanisms driving Treg proliferation during disease are poorly understood. IL-7 is a cytokine which has been recently implicated in these events [22], [27]. Thus, we measured the expression of IL-7 in the thymus and lymph nodes of WT and PACAP KO mice by real time RT-PCR. We found that the mRNA levels of IL-7 increased in the WT thymus at the peak of the disease by more than 3-fold, and decreased on day 20 (Fig. 5A). In PACAP KO mice, the expression of IL-7 was also upregulated in the thymus on day 14 after EAE. However, the mRNA levels of IL-7 were half of those in the WT mice at this time point (***P<0.001). On day 20, the differences between WT and PACAP KO mice were even more pronounced. In the lymph nodes, although mean values of IL-7 gene expression did not significantly change after MOG-treatment in any of the two mouse strains studied, PACAP KO values were significantly reduced compared to WT mice 14 and 20 days after MOG administration (Fig. 5B). We also investigated the expression of IL-2, as this cytokine has been shown to promote proliferation of Tregs in certain conditions. However, we did not find a reduction in IL-2 gene expression in the thymus or lymph nodes of PACAP KO mice (data not shown). In summary, in the absence of PACAP, alterations in IL-7, a cytokine modulating Treg physiology, may result in abnormal organ-specific generation of Tregs, which may in turn lead to enhanced disease.

### Th Profile Analysis Suggests Primarily a Reduced Th2 Response in PACAP KO Mice

**Figure 6:**
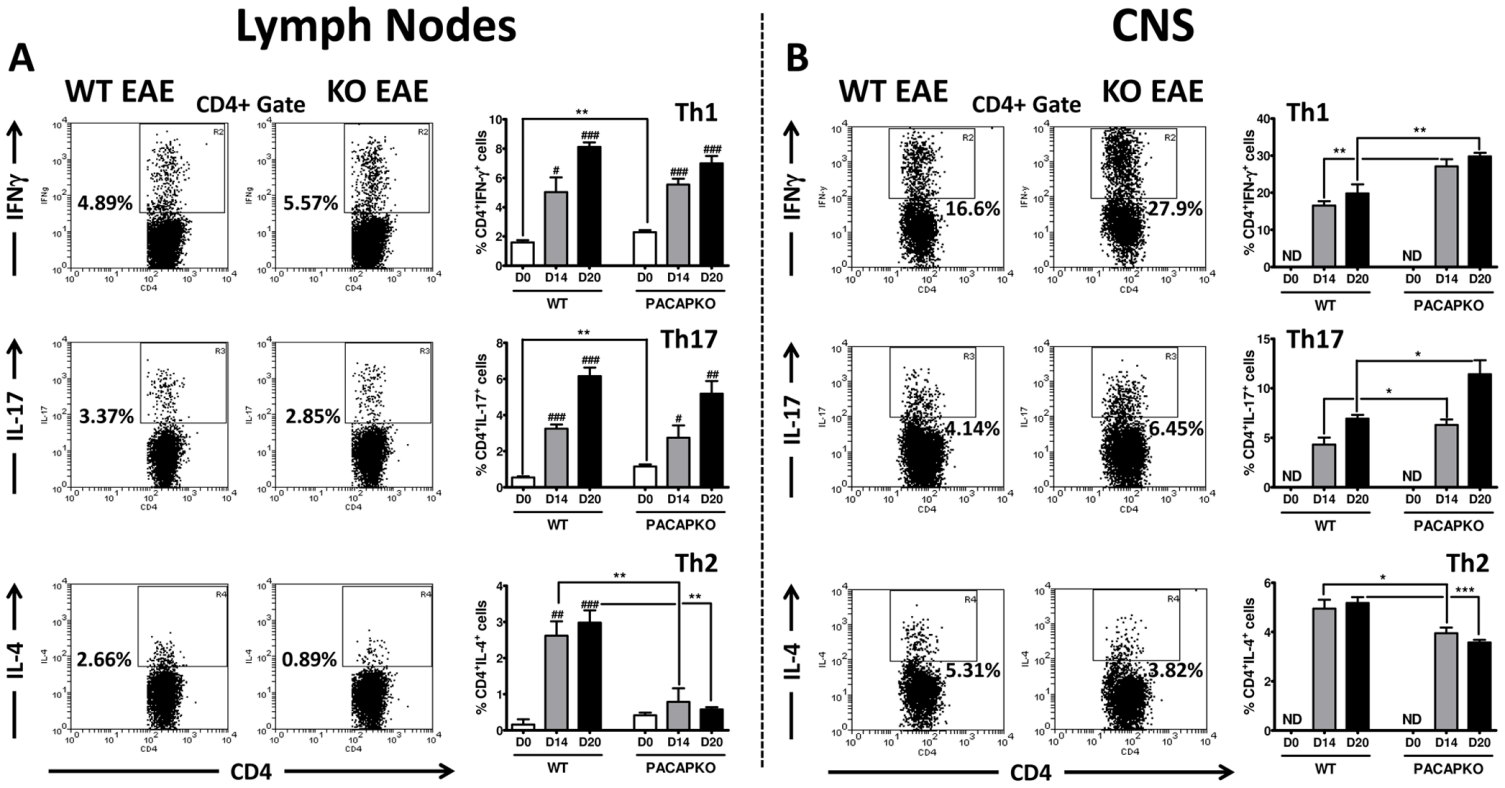
Th profile analysis in PACAP KO vs WT mice on days 0, 14, and 20 after EAE induction. Th profiles were determined in cell suspensions from the lymph nodes **(A)** and the CNS **(B),** by intracellular flow cytometry staining of IFNγ (Th1), IL-17 (Th17) and IL-4 (Th2) after 4 h incubation with PMA/ionomycin/monensin. The graphs, where Y axis represents the percentage of CD4^+^ cells that are IFNγ^+^, IL-17^+^ or IL-4^+^, respectively, are accompanied by representative FACS plots corresponding to EAE day 14. Bars represent the mean ± SEM of six individual mice. A representative experiment out of three is shown. Student’s *t*-test *P<0.05; **P<0.01, ***P<0.001 (*for comparison between values of WT and PACAP KO) and ^#^ *P*<0.05; ^##^ *P*<0.01, ^###^ *P*<0.001 (^#^for comparison between values on 0, 14 or 20 days within WT or PACAP KO mice strains).

EAE is commonly thought to be driven by IFNγ- and IL-17A-expressing CD4^+^ lymphocytes (commonly referred to as Th1 and Th17 cells) [17]. On the other hand, agents that promote Th2 responses ameliorate EAE [28], [29]. An altered balance of these T effector subsets may contribute to the enhanced susceptibility of PACAP KO mice to EAE induction [24]. We prepared cells from the draining lymph nodes and mononuclear cells from the CNS of naïve and EAE-induced WT and PACAP KO mice, cultured them for 4 hours with PMA/ionomycin/monensin, and analyzed by flow cytometry the percentages of Th1, Th2 and Th17 cells using their prototype cytokine, IFNγ, IL-4, and IL17A, respectively (Fig. 6 and Fig S2). The proportions of Th1 and Th17 cells were increased in the lymph nodes of both WT and PACAP KO mice compared to naïve mice 14 days after EAE induction (up to 3-fold compared to naïve values), with no significant differences between the two mouse strains (Fig. 6, left panels). However, the percentage of CD4^+^ cells that were Th2 was dramatically lower in EAE-immunized PACAP KO mice than in WT mice with EAE (**P<0.01) (Fig. 6, left panels). Unlike in lymph nodes, percentages of CD4^+^ cells that were Th1 and Th17 were significantly higher in the CNS in PACAP KO mice (Fig. 6, right panels). The percentages of Th1 and Th17 cells 14 days post-EAE induction were 27.1% for Th1 and 6.3% for Th17 in PACAP KO mice vs. WT, 16.5% for Th1 and 4.3% for Th17 in WT mice. Conversely, the percentage of Th2 cells was lower in PACAP KO mice compared to WT mice (average 4.0% in the KO compared to 5.0% in the WT CNS). Similar results were found in the lymph nodes and CNS 20 days after EAE induction (Fig. 6 right and left panels).

## Discussion

Inflammation is a coordinated process designed by evolution to eliminate pathogens and enable healing. However, this is carefully orchestrated in the sense that when it is no longer necessary, it must be actively terminated to avoid damage. Moreover, tolerogenic mechanisms must be in place to prevent the action of autoreactive T cells that escape negative selection. Antiinflammatory cytokines like IL-10 or TGFβ subserve some of these purposes, and are secreted by Tregs and other cells. Our prior study showing that PACAP KO mice exhibit enhanced EAE with a reduction of Tregs in the lymph nodes provided evidence that PACAP might belong to this group of intrinsic immunomodulatory molecules [24]. The current studies provide additional information on how endogenous PACAP may regulate specific T cells in this context.

In an initial set of studies, we measured PACAP gene expression in three critical organs during EAE pathogenesis: the lymph nodes, where priming of T effector cells (Teff) occurs, the CNS, the target tissue for MOG-autoreactive cells, and the thymus, the site where nTregs arise. We found that PACAP gene expression was upregulated in both the spinal cord and lymph nodes during EAE, but was not detected in the thymus. PACAP is expressed in lymphocytes [15], [16] and neurons, and it has been previously shown that injury and/or inflammation can trigger the expression of PACAP in neurons [25], [26], [30] in different experimental models *in vivo*, so the findings in the CNS and lymph nodes were not unexpected. The lack of expression in the thymus was surprising, however, because PACAP gene expression and/or PACAP immunoreactive cells were detected in the rat thymus in two prior studies [15], [16]. This likely represents a species difference. Given the induction of PACAP gene expression in the lymph nodes during EAE, it seems possible that this peptide might act in an autocrine and/or paracrine matter to regulate Treg expansion, Th priming or other aspects of adaptive immunity. In this respect, the induction of gene expression in the spinal cord was also of interest. The intermediolateral (IML) presynaptic sympathetic neurons of the spinal cord that target the thymus, lymph nodes, and other immune organ are known to express PACAP [30], [31], and are felt to be major autonomic regulators of immune function [32]. On the other hand, neuronal PACAP might be released within the CNS and modulate therein resident or invading inflammatory cells. PACAP could also be potentially produced by the inflammatory immune cells infiltrating the CNS. Finally, PACAP is reported to act as a growth and survival factor for neurons, oligodendrocytes, and other CNS cells, and may function in repair of myelin and/or axonal damage ([33], [34] and reviewed in [3], [35]).

Tregs may affect different aspects of EAE pathogenesis. For example, Tregs may act in the lymph nodes, limiting the priming phase of EAE. In this respect, it was shown that adoptively transferred Tregs migrated to the lymph nodes, protecting the mice from EAE [36]. In addition, Tregs may act in the target organ by inhibiting ongoing inflammation. In fact, the accumulation of IL-10-producing Tregs in the CNS correlates with recovery from EAE [37]. It has been shown that PACAP promotes the development of dendritic cells with a tolerogenic phenotype, which were able to induce adaptive Tregs both *in vivo* and *in vitro* [38]. Exogenous administered VIP alleviates EAE in part by increasing the generation of Tregs [39]. In our studies, we found that PACAP KO mice exhibited lower percentages of Tregs than WT mice before and after EAE induction. This suggests that endogenous PACAP may be a critical factor to maintain the Treg population during both homeostasis and disease. Although several mechanisms could be responsible for this deficiency, impaired proliferation of these cells in the absence of PACAP may be implicated by our data. An expansion of the Treg pool has been described during EAE and it has been associated with the natural resolution of the disease [20], [23]. For example, it has been shown that Tregs proliferate rapidly in the CNS during EAE induced by adoptive transfer [23]. We found here an increase of Ki67^+^ Tregs that continues at least until day 20 post-immunization. A role for the thymus as a source of Treg expansion during EAE has been also suggested, because thymectomized mice displayed enhanced EAE clinical scores with a lack of a recovery phase [22]. The percentage of Tregs in the thymus increased along the disease, which was associated with an upregulation in proliferation. Another study demonstrated the generation of Tregs *de novo* in the thymus after application of an inflammatory stimulus in the footpad of mice, which enabled tolerance induction in a mouse model of spontaneous encephalomyelitis [40]. However, the concept that Tregs expand in the thymus during autoimmune disease is still relatively new. We found that Tregs from lymph nodes, thymus and CNS exhibited a reduced rate of proliferation in PACAP KO mice compared to WT mice. Whether PACAP modulates Treg proliferation directly or indirectly through modulation of other cell type activities such as cytokine production remains to be elucidated.

The mechanisms responsible for Treg proliferation in the thymus during inflammation have only begun to be investigated. In this sense, the expression of three cytokines, IL-2, IL-15 and IL-7, which share a critical receptor subunit implicated in Treg biology [41], were analyzed in the thymus of EAE-immunized animals [22]. These are pleiotropic cytokines which modulate multiple aspects of the T cell physiology including differentiation and proliferation. A recent study showed that IL-7 (and not IL-2 or IL-15) expression increases in thymic stromal cells at the peak and recovery from MOG-induced EAE [22]. Tregs normally express low levels of CD127, the IL-7 receptor α chain [42]. However, it was shown that CD127 in these cells is highly upregulated upon EAE induction [22]. IL-7 has been associated with thymic Treg proliferation. In this sense, IL-7 induced the proliferation of thymic Tregs *in vitro* [22]. The proliferative response of Tregs to IL-7 was stronger in cells from mice with EAE compared to naïve mice. This correlates with the enhanced expression of the receptor for this cytokine after EAE. Moreover, administration of an IL-7 blocking antibody to mice with EAE resulted in reduced Treg proliferation, demonstrating the relevance of this pathway *in vivo*. In our model of EAE, we found that IL-7 increased at the peak of the disease in the thymus of WT mice. In PACAP KO mice, the induction of IL-7 was severely compromised on days 14 and 20 post-EAE induction. In contrast, IL-7 mRNA levels in the lymph nodes were not significantly increased in WT mice over the course of EAE. Despite this, IL-7 gene expression was found to be significantly reduced in the lymph nodes of PACAP KO compared to WT mice 14 and 20 days after MOG administration, suggesting that IL-7 may also be affected in the lymph nodes of PACAP KO mice. Whether PACAP modulates directly or indirectly the expression of IL-7 remains to be elucidated. We also investigated the expression of IL-2 in the KO mice, as this cytokine is commonly used *in vitro* to stimulate the proliferation of Tregs [43]–[45]. However, we failed to observe defects in the expression of this cytokine in the thymus or periphery of PACAP KO mice with EAE.

In addition to Tregs, Th effector subsets (Th1, Th17, and Th2) were found to be significantly altered in the CNS of PACAP KO mice. Th1 and Th17 cells are important players of the immune defense against pathogens [46], [47]. However, autoimmune cells of these phenotypes play a major role in the development of chronic inflammatory diseases such as MS. Whether Th1 or Th17 cells are the most critical for the development of EAE is still a matter of debate. In fact, it is now believed that both cell types may be involved in MS, but different relative proportions of them may lead to different clinical manifestations of the disease [17]. In the present study, we found that the proportions of CD4 cells that were IFNγ^+^ (Th1) and IL-17^+^ (Th17) were higher in the CNS of PACAP KO mice compared to that of WT mice. We did not find increased Th1 and Th17 in the lymph nodes of PACAP KO mice, although we previously found that *ex vivo* lymph node cultures from these mice in the presence of MOG contained higher levels of IFNγ and IL-17. The discrepancy in the latter results may be due to the different experimental approaches employed. In the cultures, comparable amounts of cells from the two mouse strains were subjected to three day stimulation with MOG, and we found increased proliferation of PACAP KO cells, which may contribute to the enhanced IFNγ and IL-17 levels found. In addition, in agreement with the enhanced inflammation in the KO mice, the relative numbers of Th1 and Th17 cells tended to be higher in PACAP KO lymph nodes.

Unlike the rather modest effect of PACAP loss on Th1 and Th17 subsets, we observed a pronounced reduction in proportions of Th2 cells both in the lymph nodes and in the CNS of PACAP KO mice. Beneficial roles for Th2 cells in EAE have been described. In this sense, certain drugs like glatiramer acetate or conditions like helminth infection that skew the Th response to Th2, abrogate EAE [28], [48]. PACAP has been shown to enhance Th2 responses *in vitro* and *in vivo* through several mechanisms of action. For example, PACAP inhibited *in vitro* the production of IL-12, a cytokine that drives Th1 differentiation, by macrophages and microglia [10], [49]–[51], and therefore might skew polarization towards Th2. In addition, it has been reported that PACAP preferentially inhibited the clonal deletion of Th2 cells, therefore promoting *in vivo* the effector function and memory phenotype of these cells [52]. In fact, PACAP improved Th2 cell survival at least in part by downregulating the expression of FasL in these cells [52].

PACAP may also modulate Th cell migration into the spinal cord or elsewhere during neuroinflammation by acting on chemokine production, which might explain the stronger differences found in the CNS Th profiles compared to the lymph nodes. In our study, we found that Th1 and Th17 cells were more abundant in the CNS of PACAP KO mice than in WT mice. Interestingly, it has been shown that PACAP decreased *in vitro* the frequency of microglia expressing the Th1-associated chemokine CXCL11 [6], which has been reported to increase in spinal cord and lymph nodes in rats with EAE [53]. In addition, PACAP elevated in the same experimental system the frequency of microglia expressing the Th2-associated chemokine CCL11, and thus may potentiate the migration of this cell subtype.

The signaling pathways by which PACAP mediates the immunomodulatory actions reported here remain to be elucidated. However, some data indicate that the signaling molecule commonly induced by PACAP, cAMP, may be involved in several aspects of innate and adaptive immunity [54], [55]. For example, elevations in intracellular cAMP were shown to suppress innate immune functions of monocytes, macrophages and neutrophils predominantly through the modulation of the generation of inflammatory mediators (e.g. cytokine, chemokine, and lipids), phagocytosis and intracellular killing of ingested pathogens. It has been also shown that a sustained elevation of cAMP levels in human resting lymphocytes induced the expression of the inhibitory molecule CTLA-4 (Cytotoxic T-Lymphocyte Antigen 4) and a suppressive phenotype in these cells [56], [57]. Moreover, studies with human and murine samples have implicated high intracellular cAMP levels in Tregs in this immunosuppressive function [58], [59]. As discussed, the closely-related neuropeptide VIP was reported to increase the production of iTregs by a mechanism that involves induction of tolerogenic dendritic cells [38]. However, that mechanism does not appear to apply to our observations in PACAP KO mice because the induction of Tregs in the C57BL/6 MOG EAE model involves expansion of the nTreg population rather than do novo production of iTregs [21].

Overall, our study demonstrates that loss of endogenous PACAP results in decreased Treg proliferation during EAE, and a major impairment in the Th2 subset, suggesting that this peptide has a critical role in the modulation of EAE pathogenesis. Furthermore, we revealed a new role for PACAP as a natural modulator of Treg expansion in the thymus during EAE, which may be indirectly mediated by IL-7.

## Supporting Information

Figure S1 Fluorescence minus one (FMO) controls for Treg gating. The following gating strategy was followed: A. Gating of CD25^+^Foxp3^+^ cells in the lymph nodes and CNS within the CD4^+^ population (left panel no CD25, center panel no Foxp3, right panel with CD25 and Foxp3 antibodies); B. Gating of Foxp3^+^Ki67^+^ cells in the lymph nodes and CNS within the CD4^+^CD25^+^ Foxp3^+^ population (left panel no Ki67 vs. right panel with Ki67 antibody); C. Gating of CD4^+^Foxp3^+^ cells in the thymus within the CD4^+^CD8^−^ population (left panel no Foxp3 vs. right panel with Foxp3 antibody); D. Gating of Foxp3^+^Ki67^+^ cells in the thymus within the CD4^+^CD8^−^Foxp3^+^ population (left panel no Ki67 vs. right panel with Ki67 antibody). Representative plots are shown out of three experiments.
(TIF)

Figure S2 Flow cytometry controls for Th gating. Unstimulated cells (no PMA and ionomycin) from the lymph nodes (left panels) and CNS (right panels) stained with CD4 and IFNγ, IL-17 or IL-4 antibodies were used to set up the gate delimiting double positive (CD4^+^cytokine^+^ ) cell populations.
(TIF)

Figure S3 PACAP KO mice exhibit exacerbated EAE. PACAP KO and C57BL6 female mice (n = 8) were immunized with MOG, and clinical symptoms of paralysis were scored as described in [24] and in Material and Methods. PACAP KO mice developed exacerbated EAE, with higher clinical scores throughout the study, and impaired recovery from the disease.
(TIF)

Figure S4 Representative FACS plots of Treg abundance and proliferation analysis in the lymph nodes. Panel A compares the proportions of Tregs 0, 14 and 20 days after EAE in the lymph nodes of WT and PACAP KO mice (n = 6 for each) using CD4, CD25 and Foxp3 antibodies (cells gated on the CD4^+^ population). Panel B compares the proportion of Tregs that are proliferating by using an additional Ki67 antibody (cells gated on the CD4^+^CD25^+^Foxp3^+^ population). Representative FACS plots are shown.
(TIF)

Figure S5 Total number of cells, % of total cells that are Tregs, and total numbers of Tregs in the lymph nodes of PACAP KO vs. WT mice. Assays were performed in six mice of each genotype on day 0, 14, day 20 after MOG administration. Panel A indicates the total numbers of cells; B the Treg percentage within the total population of cells; and C the total number of Tregs. Tregs were defined as in Figure S4 as CD4^+^CD25^+^Foxp3^+^ cells. Student’s *t*-test *P<0.05, **P<0.01 (*for comparison between values of WT and PACAP KO) and ^##^ *P*<0.01; ^###^ *P*<0.001 (^#^for comparison between values on 0, 14 or 20 days within WT or PACAP KO mice strains).
(TIF)

Figure S6 Representative FACS plots of Treg abundance and proliferation analysis in the CNS. Panel A compares the proportions of Tregs (CD4^+^CD25^+^Foxp3^+^) in the CD4^+^ population on day 14 and 20 days after EAE in the CNS of WT and PACAP KO mice (n = 6 for each) (cells gated on the CD4^+^ population). Panel B compares the proportions of Tregs that are proliferating by using an additional Ki67 antibody (cells gated on the CD4^+^CD25^+^Foxp3^+^ population) (B). Representative FACS plots are shown.
(TIF)

Figure S7 Total number of cells, % of total cells that are Tregs, and total numbers of Tregs in the CNS of PACAP KO vs. WT mice. Assays were performed in six mice of each genotype on day 14 and day 20 after MOG administration. Panel A indicates the total numbers of cells; B the Treg percentage within the total population of cells; and C the total number of Tregs (C) (n = 6 for each). Tregs were defined as in Figure S6 as CD4^+^CD25^+^Foxp3^+^ cells. Student’s *t*-test *p<0.05, ***p<0.001.
(TIF)

Figure S8 Analysis of the total numbers of thymus cells in WT vs. PACAP KO mice during EAE. The total number of cells was counted in WT and PACAP KO mice (n = 6) on days 0, 14 and 20 after EAE induction. Student’s *t*-test *p<0.05, **p<0.01.
(TIF)
