# Supplementary figures and images for: Pituitary Adenylate Cyclase Activating Peptide Deficient Mice Exhibit Impaired Thymic and Extrathymic Regulatory T Cell Proliferation during EAE
Source: PLoS One. 2013 Apr 16;8(4):e61200. doi: 10.1371/journal.pone.0061200 (PMC3628797; doi:10.1371/journal.pone.0061200)

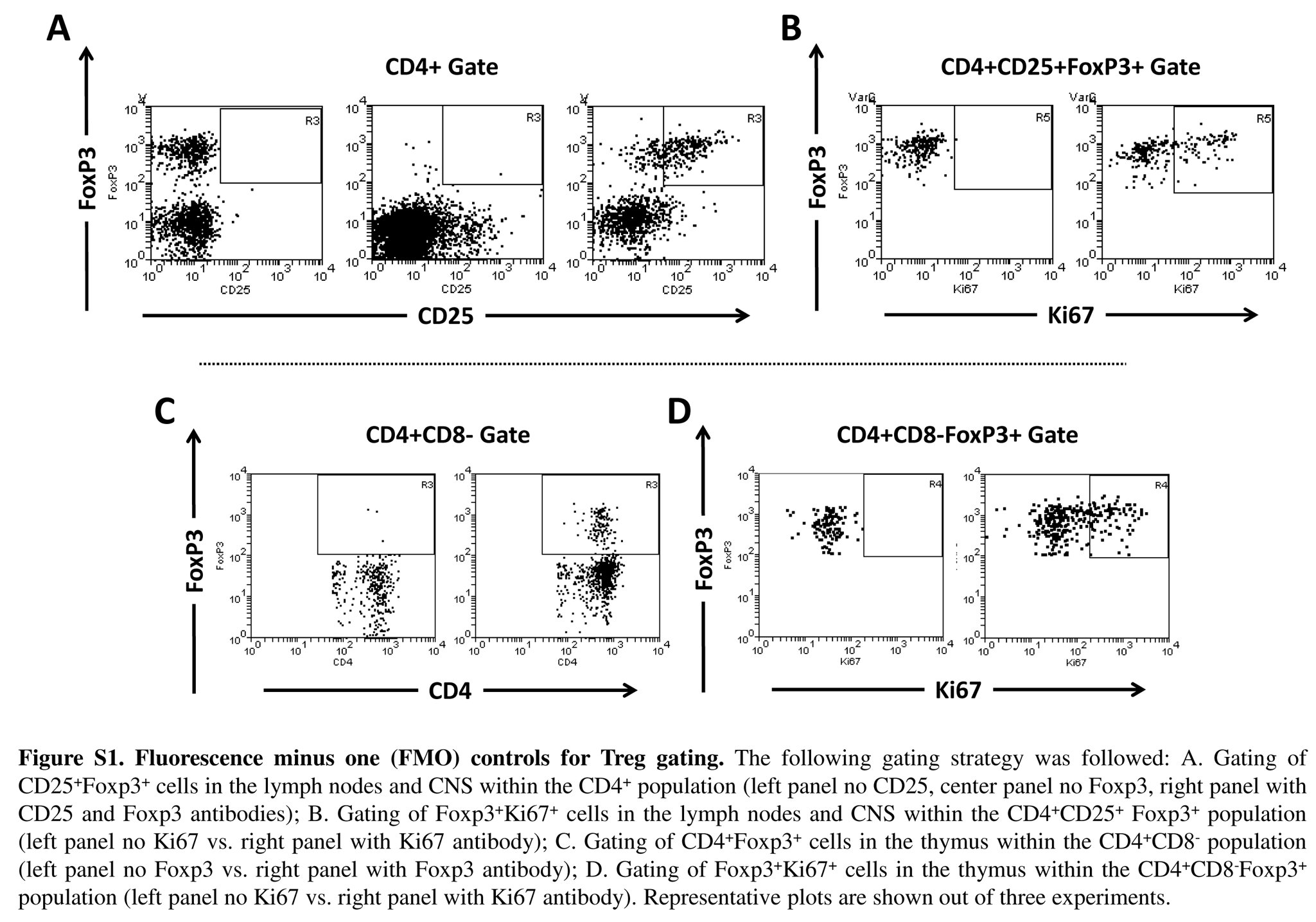

Supplement: Figure S1 — Fluorescence minus one (FMO) controls for Treg gating. The following gating strategy was followed: A. Gating of CD25+Foxp3+ cells in the lymph nodes and CNS within the CD4+ population (left panel no CD25, center panel no Foxp3, right panel with CD25 and Foxp3 antibodies); B. Gating of Foxp3+Ki67+ cells in the lymph nodes and CNS within the CD4+CD25+ Foxp3+ population (left panel no Ki67 vs. right panel with Ki67 antibody); C. Gating of CD4+Foxp3+ cells in the thymus within the CD4+CD8− population (left panel no Foxp3 vs. right panel with Foxp3 antibody); D. Gating of Foxp3+Ki67+ cells in the thymus within the CD4+CD8−Foxp3+ population (left panel no Ki67 vs. right panel with Ki67 antibody). Representative plots are shown out of three experiments. (TIF) [file pone.0061200.s001.tif]

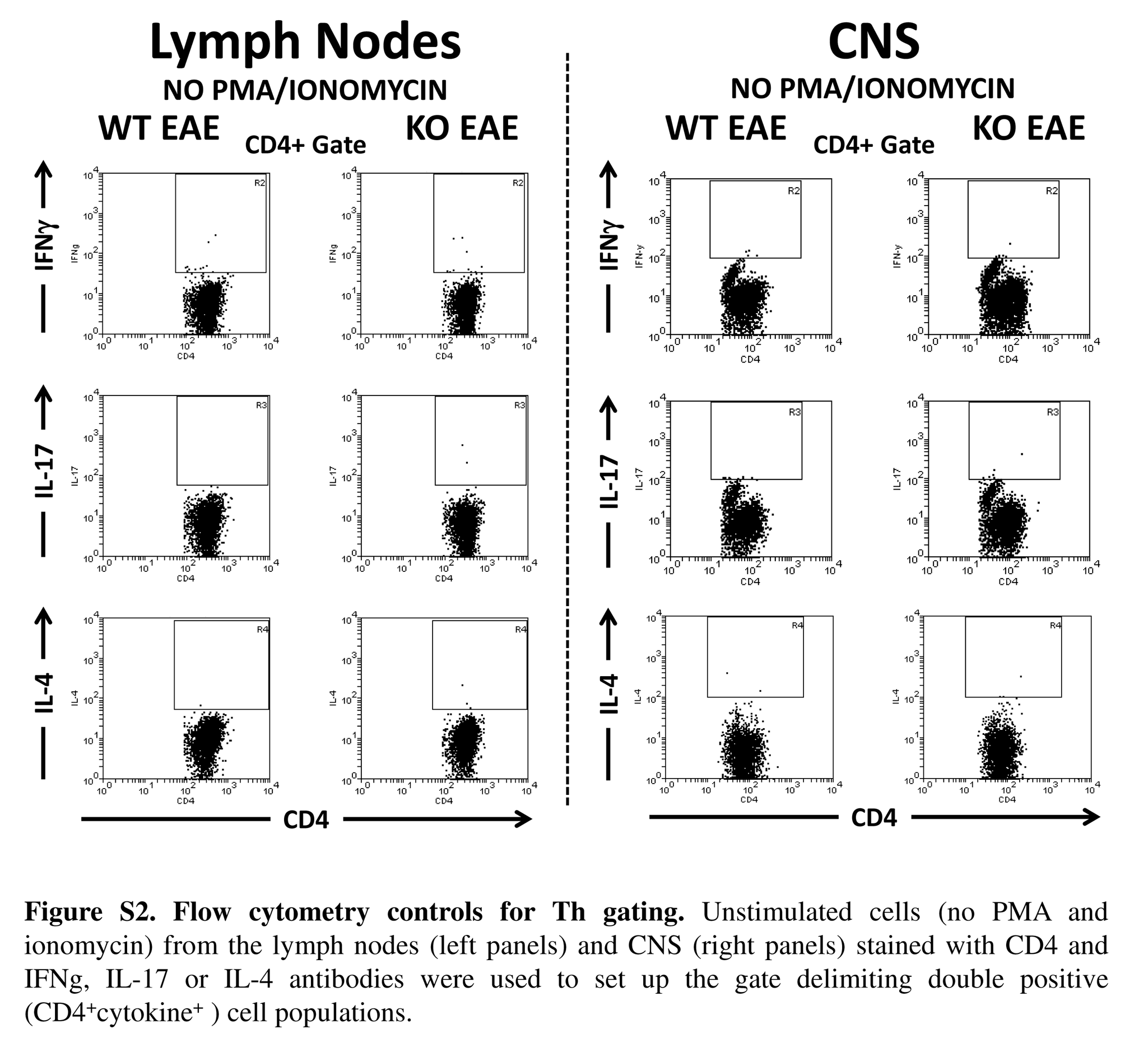

Supplement: Figure S2 — Flow cytometry controls for Th gating. Unstimulated cells (no PMA and ionomycin) from the lymph nodes (left panels) and CNS (right panels) stained with CD4 and IFNγ, IL-17 or IL-4 antibodies were used to set up the gate delimiting double positive (CD4+cytokine+ ) cell populations. (TIF) [file pone.0061200.s002.tif]

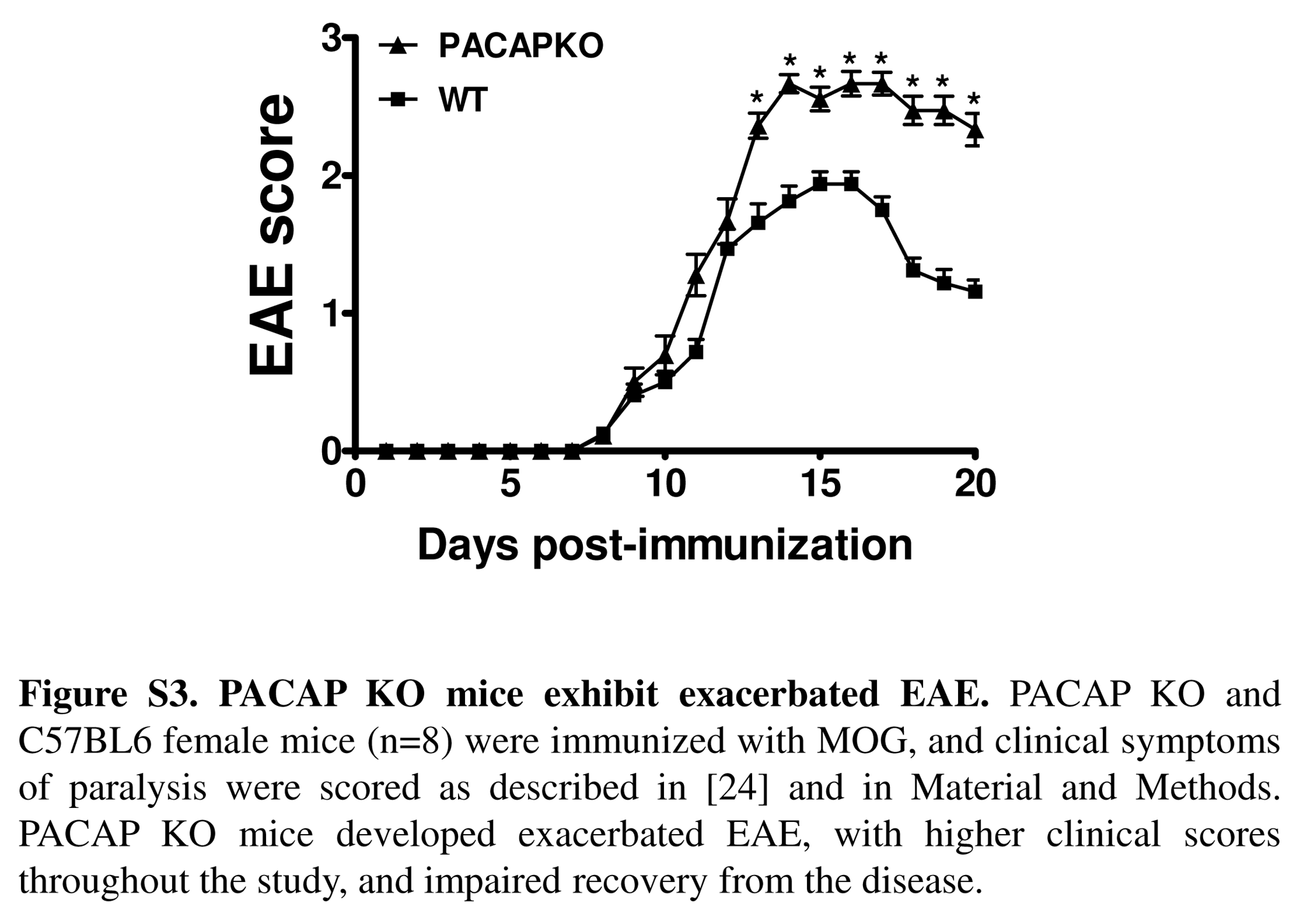

Supplement: Figure S3 — PACAP KO mice exhibit exacerbated EAE. PACAP KO and C57BL6 female mice (n = 8) were immunized with MOG, and clinical symptoms of paralysis were scored as described in [24] and in Material and Methods. PACAP KO mice developed exacerbated EAE, with higher clinical scores throughout the study, and impaired recovery from the disease. (TIF) [file pone.0061200.s003.tif]

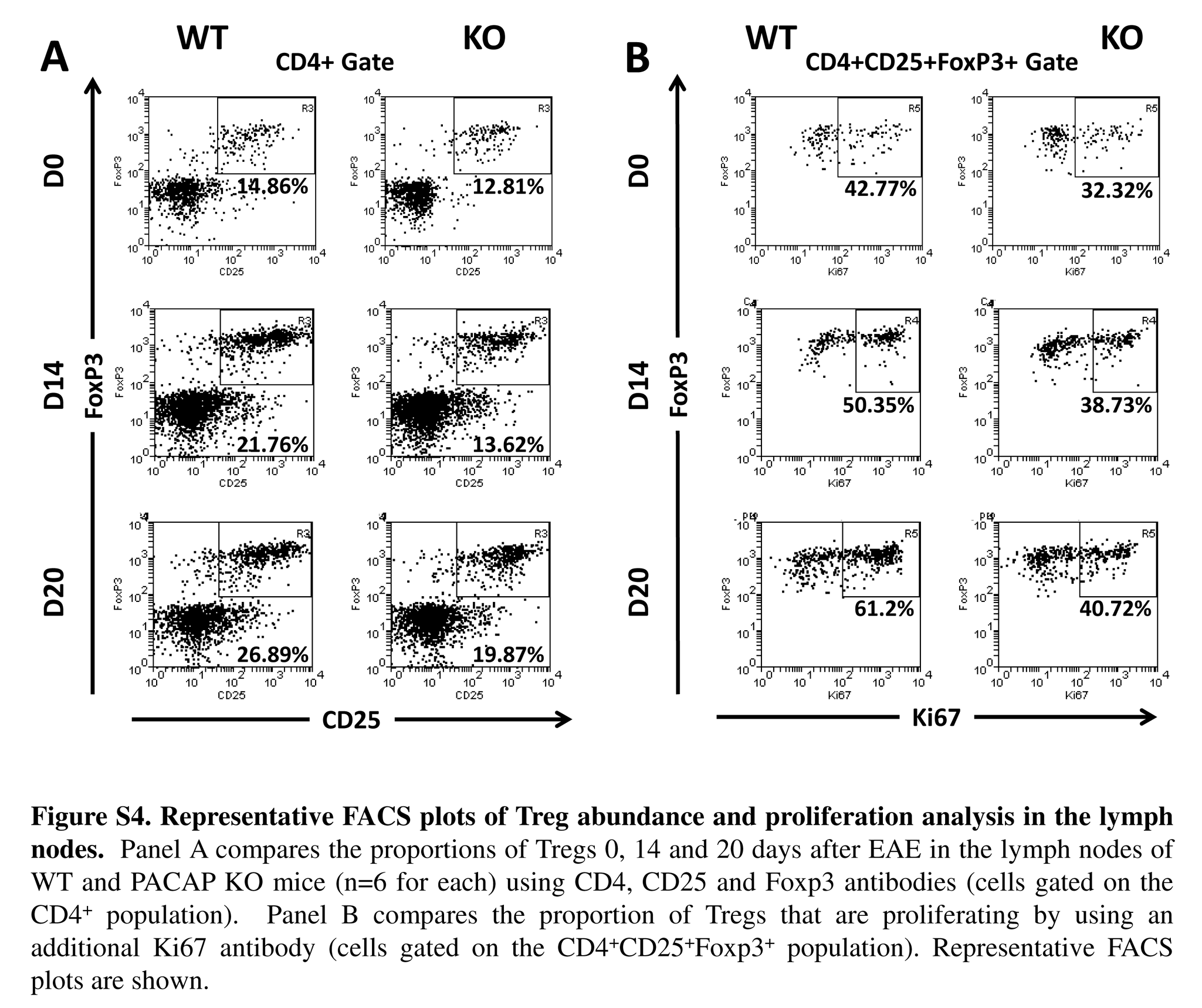

Supplement: Figure S4 — Representative FACS plots of Treg abundance and proliferation analysis in the lymph nodes. Panel A compares the proportions of Tregs 0, 14 and 20 days after EAE in the lymph nodes of WT and PACAP KO mice (n = 6 for each) using CD4, CD25 and Foxp3 antibodies (cells gated on the CD4+ population). Panel B compares the proportion of Tregs that are proliferating by using an additional Ki67 antibody (cells gated on the CD4+CD25+Foxp3+ population). Representative FACS plots are shown. (TIF) [file pone.0061200.s004.tif]

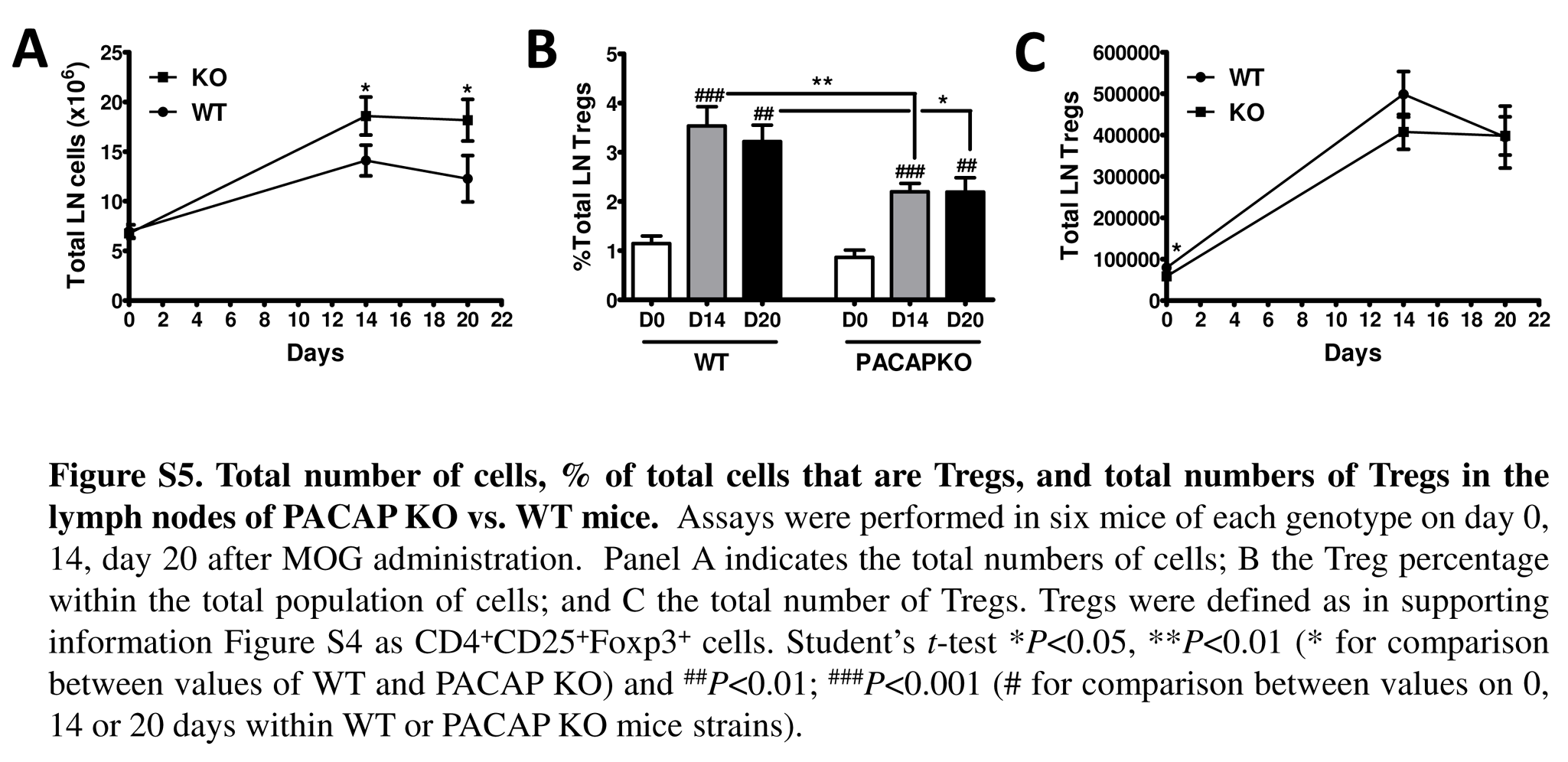

Supplement: Figure S5 — Total number of cells, % of total cells that are Tregs, and total numbers of Tregs in the lymph nodes of PACAP KO vs. WT mice. Assays were performed in six mice of each genotype on day 0, 14, day 20 after MOG administration. Panel A indicates the total numbers of cells; B the Treg percentage within the total population of cells; and C the total number of Tregs. Tregs were defined as in Figure S4 as CD4+CD25+Foxp3+ cells. Student’s t-test *P<0.05, **P<0.01 (*for comparison between values of WT and PACAP KO) and ## P<0.01; ### P<0.001 (#for comparison between values on 0, 14 or 20 days within WT or PACAP KO mice strains). (TIF) [file pone.0061200.s005.tif]

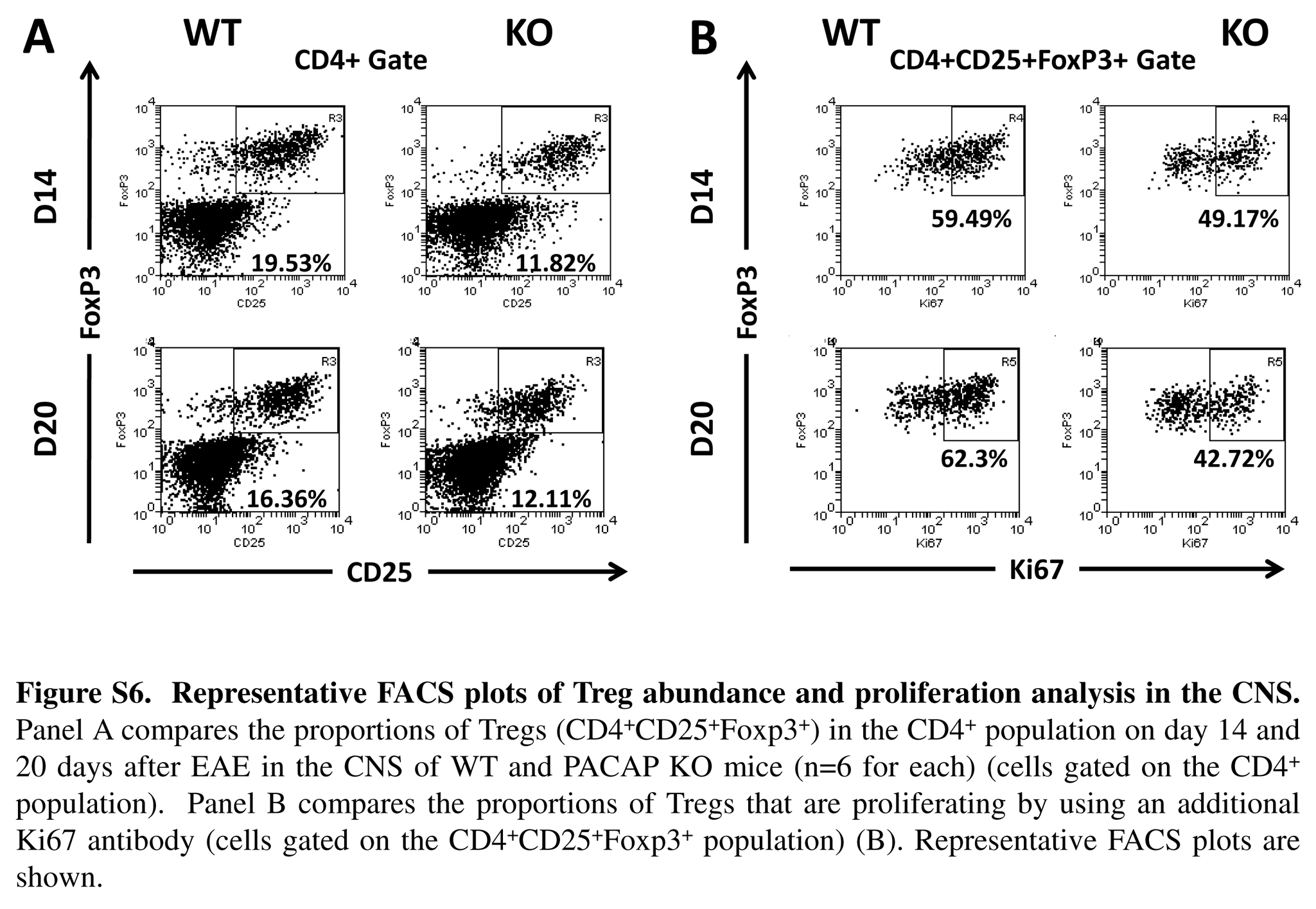

Supplement: Figure S6 — Representative FACS plots of Treg abundance and proliferation analysis in the CNS. Panel A compares the proportions of Tregs (CD4+CD25+Foxp3+) in the CD4+ population on day 14 and 20 days after EAE in the CNS of WT and PACAP KO mice (n = 6 for each) (cells gated on the CD4+ population). Panel B compares the proportions of Tregs that are proliferating by using an additional Ki67 antibody (cells gated on the CD4+CD25+Foxp3+ population) (B). Representative FACS plots are shown. (TIF) [file pone.0061200.s006.tif]

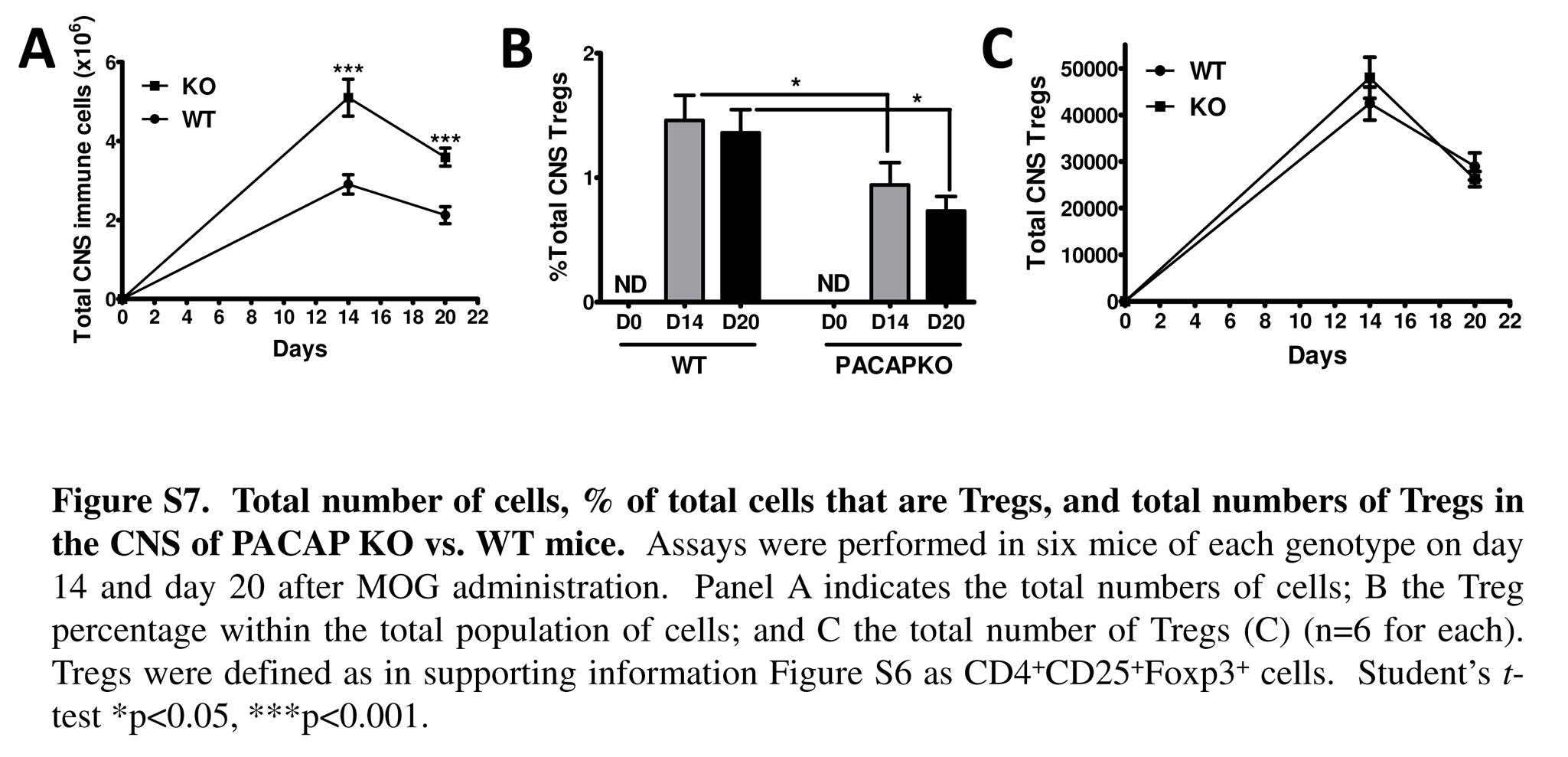

Supplement: Figure S7 — Total number of cells, % of total cells that are Tregs, and total numbers of Tregs in the CNS of PACAP KO vs. WT mice. Assays were performed in six mice of each genotype on day 14 and day 20 after MOG administration. Panel A indicates the total numbers of cells; B the Treg percentage within the total population of cells; and C the total number of Tregs (C) (n = 6 for each). Tregs were defined as in Figure S6 as CD4+CD25+Foxp3+ cells. Student’s t-test *p<0.05, ***p<0.001. (TIF) [file pone.0061200.s007.tif]

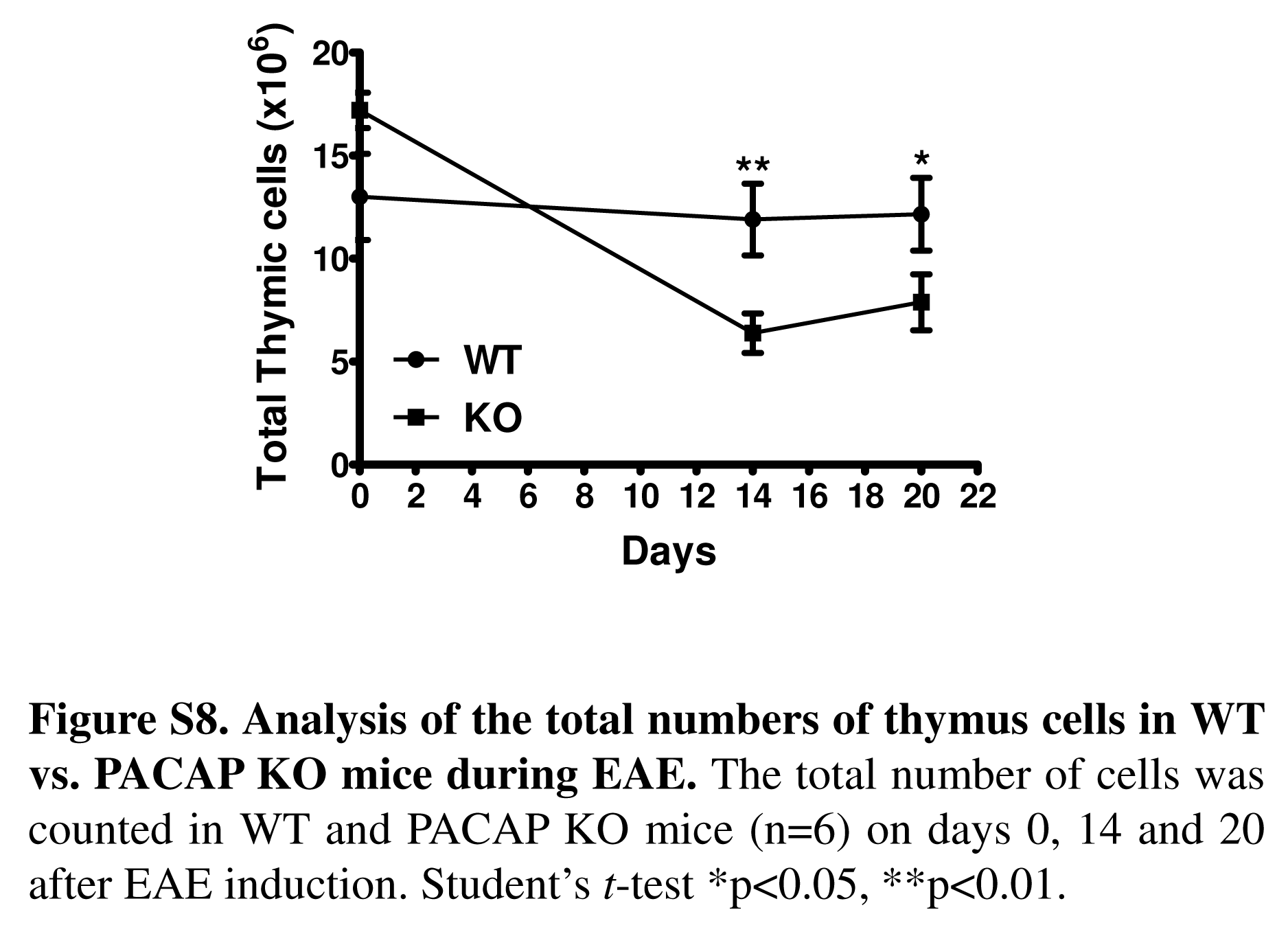

Supplement: Figure S8 — Analysis of the total numbers of thymus cells in WT vs. PACAP KO mice during EAE. The total number of cells was counted in WT and PACAP KO mice (n = 6) on days 0, 14 and 20 after EAE induction. Student’s t-test *p<0.05, **p<0.01. (TIF) [file pone.0061200.s008.tif]
